# Increases in GFAP immunoreactive astrocytes in the cerebellar molecular layer of young adult CBA/J mice

**DOI:** 10.1186/s42826-021-00100-5

**Published:** 2021-08-28

**Authors:** Cheryl Tyszkiewicz, Ingrid D Pardo, Hayley N Ritenour, Chang-Ning Liu, Chris Somps

**Affiliations:** 1 Comparative Medicine, Worldwide Research, Development and Medical, Pfizer Inc, MS 8274-1359, PGRD, Eastern Point Road, Groton, CT 06340 USA; 2 Global Pathology and Investigative Toxicology, Pfizer Inc, Groton, CT 06340 USA

**Keywords:** CBA/J mice; cerebellum, Astrocyte, Bergmann glia, Glial fibrillary acidic protein

## Abstract

**Background:**

CBA/J mice are standard experimental animals in auditory studies, and age-related changes in auditory pathways are well documented. However, changes in locomotion-related brain regions have not been systematically explored.

**Results:**

We showed an increase in immunoreactivity for glial fibrillary acidic protein (GFAP) in the cerebellar molecular layer associated with Purkinje cells in mice at 24 weeks of age but not in the younger mice. Increased GFAP immunoreactivity appeared in the form of clusters and distributed multifocally consistent with hyperplasia of astrocytes that were occasionally associated with Purkinje cell degeneration. Three out of 12 animals at 16 and 24 weeks of age exhibited pre-convulsive clinical signs. Two of these 3 animals also showed increased GFAP immunoreactivity in the cerebellum. Rotarod behavioral assessments indicated decreased performance at 24 weeks of age.

**Conclusions:**

These results suggest minimal to mild reactive astrocytosis likely associated with Purkinje cell degeneration in the cerebellum at 24 weeks of age in CBA/J mice. These findings should be taken into consideration prior to using this mouse strain for studying neuroinflammation or aging.

**Supplementary Information:**

The online version contains supplementary material available at 10.1186/s42826-021-00100-5.

## Background

CBA/J mice have been widely used in studies of immunology/inflammation, metabolism, infectious diseases, and fetal development. In neuroscience research, the CBA/J mouse has become a standard experimental animal in auditory and hearing loss studies [1–4]. This strain has also been crossed with C57BL/6 mice to generate a Huntington’s disease model [5, 6]. Interestingly, compared with other commonly used inbred mouse strains, CBA/J mice show some functional neurological deficits. For instance, it has been demonstrated that, at 10–12 weeks of age, the animals have learning deficits in the Morris water maze and the circular platform maze behavioral tests [7]. In addition, at 9 weeks of age, these mice have increased sensitivity to tonic seizures induced by administration of caffeine compared with the SWR strain of mice [8]. Bernard-Hélary and co-workers [9] found that CBA/J mice, at 12 weeks of age, also have higher susceptibility to methionine sulfoximine-induced seizure than C57BL/6J mice. It has been shown that some striatal dopaminergic functions are impaired [10] and there are fewer numbers of both dopamine cells and receptors [11] in CBA/J mice relative to BALB/c and C57BL/6J mice, though the levels of striatal dopamine as well as its metabolite were significantly higher in the CBA strain than in the BALB/c strain [12]. In the majority of studies, either young (< 10 weeks of age) or old (> 72 weeks) CBA/J mice were used to identify age-related changes in auditory pathways of the nervous system [1, 13–15]. While changes in auditory pathways in the brain have been well documented across the lifespan of this strain, locomotion-related brain areas have not been systematically evaluated. In view of this, we have examined gray and white matter as well as glial cells in two brain sections: (1) at the level of the striatum/corpus callosum and motor cortex and (2) at the level of cerebellum/medulla oblongata of CBA/J mice at 8 to 24 weeks of age using combined histologic, immunohistochemistry (ICH) and behavioral assessments.

## Results

### Histopathologic examination of the striatum, corpus callosum and motor cortex

There were no light microscopic findings in these structures in sections stained with hematoxylin and eosin (H&E, Figure S1) or glial fibrillary acidic protein (GFAP) immunochemistry (IHC, Figure S2) in any animals.

### Histopathologic examination of the cerebellum and medulla oblongata

**Fig. 1:**
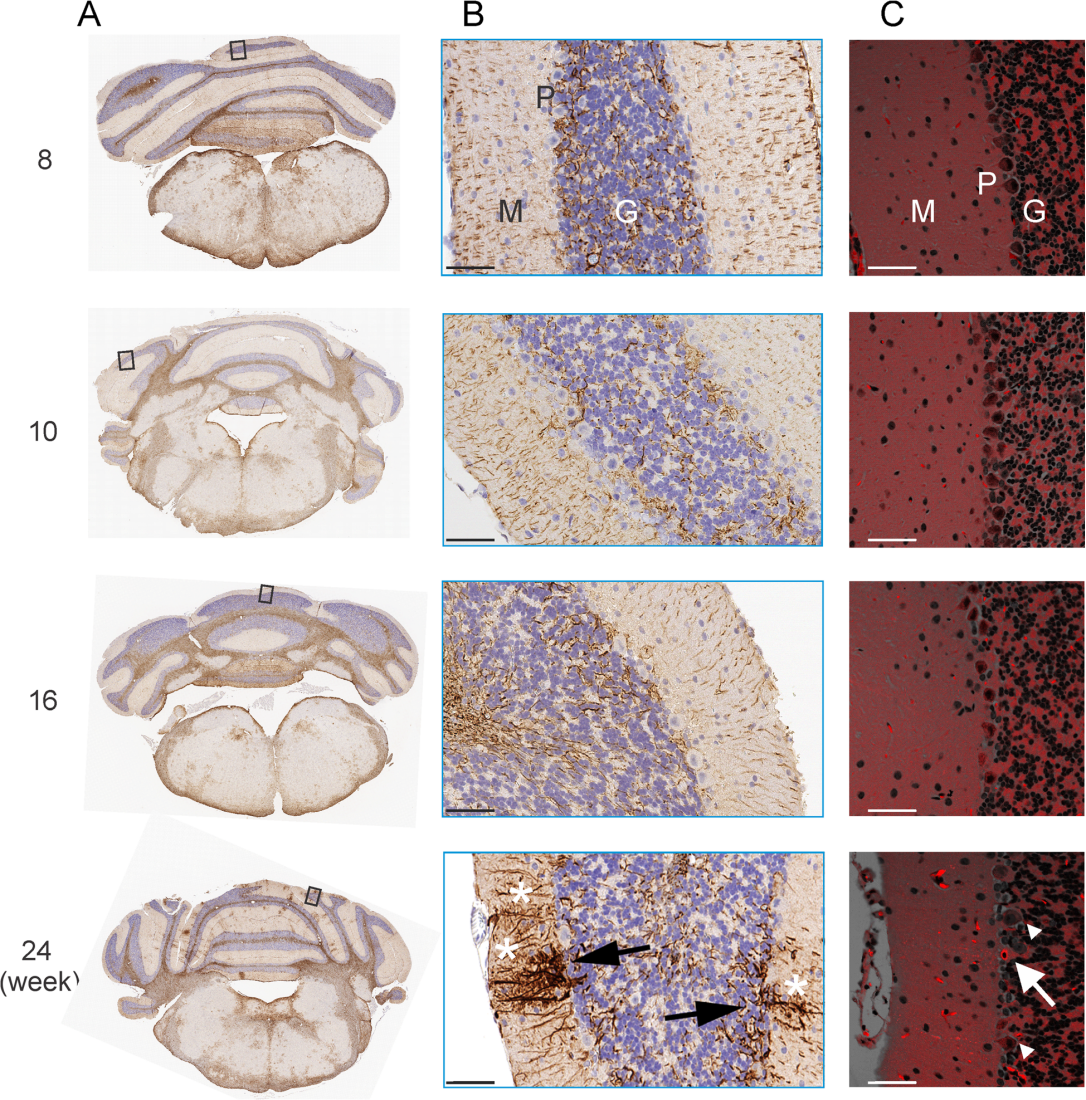
Brain sections at the level of mid-cerebellum and medulla oblongata. **A** representative IHC images of GFAP immunoreactivity in the adult male CBA/J mouse cerebellum. Increased GFAP reactivity was observed in the molecular layer of the cerebellum in mice 24 weeks of age, but not animals 8, 10 or 16 weeks of age. **B** Magnified IHC images of the corresponding boxed areas in (**A**). Increased GFAP expression in the Bergmann glia (black arrows) along Purkinje cell spines extending across the molecular layer (M) of the cerebellar cortex and terminating at the pial surface (*). **C** Representative images of the mouse cerebellum under a confocal fluorescence microscope. The images were from different H&E sections of the same animals as used for (**A**) and (**B**). The white arrow points to red eosin fluorescence indicating Purkinje cell (P) soma undergoing degeneration. Note the soma shape change from flask-like (as seen in adjacent neurons, indicated by arrowheads) to an elliptical shape (indicated by the arrow). G: Granular cell layer. Scale bar = 50 μm

There were no histopathologic findings in H&E-stained sections. GFAP IHC evaluation of mid cerebellar sections revealed multifocal, minimal to mild increased immunoreactivity for GFAP in the molecular layer of the cerebellum in 4 out of 6 animals at 24 weeks of age (Table 1; Fig. 1A&B). This increased incidence of GFAP reactivity was statistically significant at week 24 (*P* < 0.05, compared with the 3 younger age groups combined). To determine if the GFAP reactivity might be associated with Purkinje cell (PC) degeneration we conducted confocal fluorescent microscopic evaluation of H&E sections from a 24-week-old animal with increased GFAP staining. This demonstrated multifocal fluorescence of dendrites and less commonly fluorescence in the cell body of PCs (Fig. 1C). This technique reveals neurons undergoing degeneration or necrosis similar to Fluoro Jade b/c stains [16].

**Table 1:** GFAP reactivity in the molecular layer of cerebellum

Age in weeks	Unremarkable	Minimal to mild	*P* value
8	6	0	-
10	6	0	1.00
16	6	0	1.00
24	2	4	< 0.05*

*Chi^2^ test of incidence in week 24 vs. the pooled incidence of weeks 8, 10 and 16

### Rotarod and open field tests

**Fig. 2:**
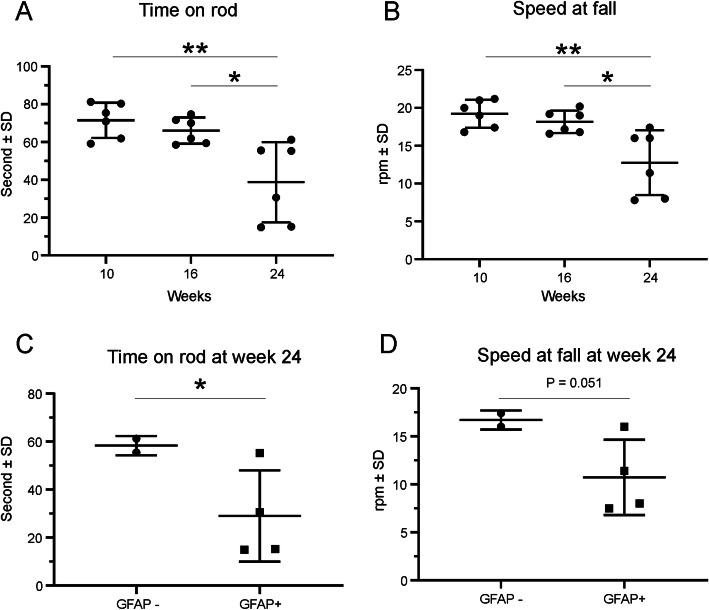
Rotarod performance of CBA/J mice at 10–24 weeks of age. Accelerating rotarod trials at 10, 16 and 24 weeks of age show a decrease in performance at 24 weeks. At 24 weeks, both the time (**A**) on the rod and the speed at falling (**B**) were decreased significantly, compared with the first 2 time points (*P* < 0.01 (**) − 0.05 (*), One-way ANOVA, *n* = 6). At 24 weeks, the rotarod performance was decreased significantly in those animals with activated GFAP in the cerebellum (C&D, *: *P* ≤ 0.05, unpaired *t*-test)

Since the cerebellum plays an important role in coordinated movement, motor learning and vestibular function, and cerebellar damage results in impaired body balance and disturbance in gait and posture, we tested animals’ coordination behavior on the rotarod. Mice at 24 weeks of age showed decreased time on the rotarod (Fig. 2A) and fell off the rotarod at lower speeds (Fig. 2B) when compared with younger animals. (*P* < 0.05 − 0.01, one-way ANOVA). Interestingly, at week 24 the 4 animals with increased GFAP expression had significantly shorter durations on the rotarod and fell at lower rod velocities (Fig. 2C&D, *P* < 0.05 and *P* = 0.05, respectively, *t*-test). There were no age-related differences observed in the open field locomotion tests (2486, 2488, and 2802 beam breaks at weeks 10, 16, and 24, respectively, *P* > 0.05).

### Seizure

All animals appeared normal in weeks 8 and 10 with Racine scores between 1 and 4. However, at week 16, 1 out of 6 animals showed a score of 5 on the Racine scale (Table 2). In week 24, 2 out of 6 animals showed seizure grades above a Racine score of 5. However, none of the animals exhibited full convulsions (≥ Racine score of 6). Interestingly, in the 24-week old group, both the animals with the Racine score of 5 had increased GFAP reactivity in the cerebellum (Table 2).

**Table 2:** Racine score and incidence

Age in weeks	Score 0	Score 1–4	Score 5 & above
8	6	0	0
10	6	0	0
16	5	0	1
24	4	0	2^a^

^a^Both animals showed increased GFAP expression in the cerebellum

**Table 3:** Racine score behavioral responses

Racine Score	Behavioral Responses	Description
1	No behavioral change	Subject appears normal
2	Facial clonus	Facial and oral activity that may include ipsilateral eye closure and blinking
3	Head nodding	Head nodding, head bobbing, tongue chewing, chewing like a cow, drooling, wet dog shakes, tremor, body jerks/twitches
4	Unilateral forelimb clonus	One forelimb stretches out uncontrollably
5	Rearing and bilateral forelimb clonus	Both forelimbs stretch out uncontrollably, extended body posture and straight erect (Straub) tail can be seen, rearing occurs, retropulsion
6	Rearing and falling	Rearing and falling, may happen in quick succession (continuous), loss of postural control
7	Full seizure	Could be clonic, or clonic-tonic-clonic seizures involving all 4 limbs

## Discussion

In this study we observed an increase in reactive astrocytes by GFAP immunoreactivity in the molecular layer of the cerebellum in CBA/J mice at 24 weeks of age, compared with mice at 16 weeks of age and younger. There were no similar increases in reactive astrocytes in other regions of the cerebellar cortex or other brain regions examined, including motor cortex, striatum and corpus callosum, nor any lesions observed with H&E examination. Notably, 24-week-old mice also did not perform as well on the rotarod tests as the younger animals, especially those mice that showed increased reactive astrocytes.

Astrocytes carry out a wide-ranging set of activities including neurotransmitter uptake, synthesis and secretion of trophic factors, aid in repair and regeneration of wounds and regulation of synaptic density [17]. Moreover, astrocytes exhibit a high degree of plasticity and they are able to modify their morphology and function throughout life [18]. GFAP is an intermediate-filament protein [19] and is one of the hallmarks of astrocytic differentiation and functional activity during development and aging [20]. Although generalized increases in GFAP expression have been reported with aging throughout the rodent CNS [21, 22], these increases are typically observed in C57BL/6J mice older than 24 weeks and are not restricted to the cerebellum. GFAP increases are also observed with CNS injuries and neurodegeneration, and are believed to represent a restorative response by astrocytes [23].

In the molecular layer of the cerebellar cortex, specialized astrocytes called Bergmann glial cells are thought to insulate, maintain and regulate the Purkinje cell (PC) neuronal structure and function in the adult cerebellum. For example, the experimentally induced ablation of Bergmann glial cells in transgenic mice causes severe defects of cerebellar neurons as well as motor discoordination [24]. Additionally, in the PC degeneration mutant mice, degeneration of cerebellar PCs is associated with activated Bergmann glia in the molecular layer of the cerebellum [25]. Thus, increased GFAP immunostaining in our 24-week-old CBA/J mice may reflect Bergmann cell activation in response to injury to the PCs.

The cerebellum is involved in motor learning and coordination, and it controls voluntary learned physical movements. It is well documented that cerebellar PCs are involved in animal’s motor coordination [26–28]. Locomotive function of the Purkinje cell degeneration mutant mice was impaired in rotarod test [29, 30]. Zhang and coworkers [29] also showed that fetal graft implantation of healthy cerebellum (into cerebellar hemispheres) can correct the motor impairment in balance and coordination as measured in the rotarod test at 6 weeks of age. The decreases in performance of the 24-week-old CBA/J mice on the rotarod in the current study thus suggests there may be injury to the cerebellar PCs in these animals. This is indeed consistent with our observation of PC degeneration in the cerebellum of the 24-week-old animal examined using the H&E autofluorescence technique (Fig. 1C) as the the animals that showed increased GFAP immunoreactivity also performed poorly in the rotarod test. It is unlikely that the decreased rotarod performance was due to body weight or size increases between 16 and 24 weeks, since the rotarod speeds and accelerations we used are typical for young adult mice [31].

Consistent with reports that CBA/J mice might have lower seizure thresholds than other strains, like C57BL/6 mice [9], and sexually mature CBA males may be more susceptible than mature females [32], we observed seizure-like clinical signs in mature male CBA/J mice at 16–24 weeks of age. These included seizure-like behavioral scores of 5 or 6 on a 7-point modified Racine scale [33–35]. Although we did not observe overt convulsions during the day, nocturnal mice are suspected to have seizures more often at night (personal communication with Robert Garman) during the animals’ more active period [36]. However, in the current study we did not make any observations during the night (i.e., lights off) period. Epilepsy and seizure are known to be associated with cerebellar atrophy and PC degeneration in humans [37, 38] and animals, and are believed to result from excitotoxicity and PC Ca^2+^ overload [39]. PC degeneration was observed with MRI in patients with generalized clonic/tonic seizures [40] and in postmortem evaluations of patients with status epilepticus [41]. In a patient with prolonged status epilepticus there was PC loss, injury to the remaining PCs, and increased prominence of the Bergmann glia [42]. Seizure-susceptible gerbils showed reduced PC counts attributed to seizure in these animals [43]. Intracerebroventricular injections of excitatory neurotransmitters glutamate and aspartate just rostral to the hippocampus of rats caused seizures, neuronal loss and reactive gliosis in the hippocampus, along with PC cell necrosis in the cerebellum [44].

Although we cannot definitively conclude that the astrocytosis we observed in the cerebellar molecular layer of 24 week old male CBA/J mice is the result of seizures, it is notable that mice in this age category did score higher on the Racine seizure scale, showed evidence of PC necrosis and performed worse on the rotarod motor response test than younger animals.

## Conclusions

We have shown increased GFAP reactivity in the cerebellum and behavioral changes in 24-week-old male CBA/J mice. Therefore, these factors should be considered when this strain and sex of animals is to be used in neuroinflammation or aging studies.

## Methods

### Animal care and study design

Twenty-four (24) male CBA/J mice (7–8 weeks) were purchased from the Jackson Laboratory (Bar Harbor, ME). All activities involving animals were carried out in accordance with federal, state, local and institutional guidelines governing the use of laboratory animals in research and were reviewed and approved by Pfizer’s Institutional Animal Care and Use Committee (IACUC).The animals were group-housed (*n* = 3/cage) in an AAALAC-accredited vivarium with room temperature of 20–26 °C and humidity of 30–70 %, under a 12 h:12 h light–dark cycle, and had *ad libitum* water and a regular mouse chow (5002, PMI Feeds Inc. Richmond, IN). Six mice were assigned to be investigated and euthanized at 8, 10, 16 and 24 weeks of age. Eight-week-old animals were euthanized without any behavioral assessments being performed whereas the remaining animals in the 10, 16, and 24-week-old groups underwent behavioral testing prior to the scheduled necropsies. Animals were acclimated for 5 days before experimental procedures were started. To mimic routine animal handling and dosing procedures, all animals were injected intraperitoneally with saline (10 mL/kg) every other day for receiving a total of 3 injections starting at 8 weeks of age prior to behavioral tests being performed.

### Rotarod test

At the ages of week 10, 16 and 24, 6 mice were evaluated for balance and coordination on a rotarod system (AccuScan Instruments, Inc., Columbus, OH) [45, 46]. Mice were acclimated to the rotarod apparatus for 2 days before each experimental session. Each acclimation period consisted of placing the animals on the rotarod for approximately 120 s at a constant rate of 5 revolutions per minute (RPM). During the test day, mice were acclimated to the testing room for approximately 10 min before beginning of the procedure. Testing began at a speed of 5 RPM for 60 s as stabilization period. The speed was then accelerated up to 40 RPM at a rate of 0.2 revolutions per second for an additional 175 s, or until the mouse fell off the rod. The latency to fall from the rod and speed at fall were recorded for analysis.

### Locomotor activity assessments

On different days than the rotarod testing at weeks 10, 16 and 24, the same 6 mice were tested for locomotor activity [31, 47], which included an assessment of horizontal (X-Y ambulation) movements for a total session length of 30 min in a novel environment using an SmartFrame open field photobeam monitoring system (Kinder Scientific, Poway, CA). Total number of infrared beam breaks was recorded for analysis. All behavioral tests were conducted during the light phase of light-dark cycle.

### Seizure evaluation

At weeks 10, 16 and 24, the same 6 mice were evaluated on a 7-point Racine scale adapted from a 5-point scale [33] (see Table 3). The seizure potential was rated on the same day as other behavioral testing.

### Hematoxylin-eosin staining and eosin fluorescence

At week 8 and following each behavioral testing in weeks 10, 16 and 24, 6 mice were deeply anesthetized with isoflurane and euthanized by exsanguination. The skull was rapidly removed, and the brain was carefully removed, sliced in half coronally, then fixed in cold 4 % methanol-free formaldehyde overnight. On the following day, the specimens were trimmed coronally at the level of striatum, corpus callosum (CC) and motor cortex and at the level of the mid-cerebellum and medulla oblongata [levels 2 and 6 of [48]]. The two most rostral sections of each brain level were processed and embedded into the same paraffin block. For all animals (6/age group), a 5 μm section was taken for standard H&E staining and subsequent light microscopic examination. For selected animals, eosin fluorescence evaluation [16, 49] using a confocal microscope was performed (Zeiss LSM 880 Airyscan, Carl Zeiss AG, Oberkochen, Germany).

### Immunohistochemistry

The adjacent 5 μm section, taken from the same paraffin block that was used for H&E staining, was used for GFPA IHC. The slides were heat-treated for 20 min for antigen retrieval, and then blocked with BOND Polymer Refine Detection Kit (Leica Biosystem, Germany) for 5 min and followed in Dako protein block solution for 10 min. Sections were stained with rabbit polyclonal anti-GFAP antibody (1:2000, #Z0334, Dako, Agilent Technologies Inc, Santa Clara, CA) for 15 min. The sections were then rinsed and incubated in appropriate rabbit-specific secondary antibodies, which were tagged with chromogen in the BOND Polymer Refine Detection Kit. Rabbit IgG (I-1000, 1:3500, Vector Laboratories, Burlingame, CA) was used as the negative control. All the processes were carried out by a Leica Bond-RX Autostainer 002. Microimages were captured using LAS X (Leica Application Software) at 20X.

### Statistical analysis

All the results are reported as mean ± standard deviation (SD). The data were statistically analyzed by Student’s *t*-test, or one- or two-way analysis of variance (ANOVA) followed by Dunnett’s or Bonferroni’s post hoc test using Graphpad Prism 8 software (La Jolla, CA). Chi square test was performed using the on-line statistical application [50]. *P* values of less than 0.05 (*) were considered statistically significant.

## Supplementary Information

**Additional file 1: Figure S1.** Brain sections at the level of the striatum, corpus callosum and motor cortex stained with H&E from animals in various ages as shown on the left of the images. There were no findings from any regions shown.

**Additional file 2: Figure S2.** Brain sections at the level of the striatum, corpus callosum and motor cortex. (A) representative IHC images of GFAP immunoreactivity in adult male CBA/J mouse forebrain. No increased GFAP reactivity was observed in any of the areas mentioned above from mice of any age. (B) Magnified IHC images of the corresponding boxed areas in the cortex (A). Unlike the GFAP expression in the cerebellum, no increased GFAP expression was observed in the superficial layers of the cortex at age of week 24. Scale bar = 60 µm.

## Data Availability

The datasets generated and/or analyzed during the current study are available from the corresponding author upon request.

## Abbreviations

AAALAC: The Association for Assessment and Accreditation of Laboratory Animal Care International
CC: Corpus callosum
CNS: Central nervous system
GFAP: Glial fibrillary acidic protein
ICH: Immunohistochemistry
IACUC: Institutional Animal Care and Use Committee
H&E: Hematoxylin and eosin
PC: Purkinje cell
RPM: Revolutions per minute
