# Supplementary figures and images for: Increases in GFAP immunoreactive astrocytes in the cerebellar molecular layer of young adult CBA/J mice
Source: Lab Anim Res. 2021 Aug 28;37:24. doi: 10.1186/s42826-021-00100-5 (PMC8400896; doi:10.1186/s42826-021-00100-5)

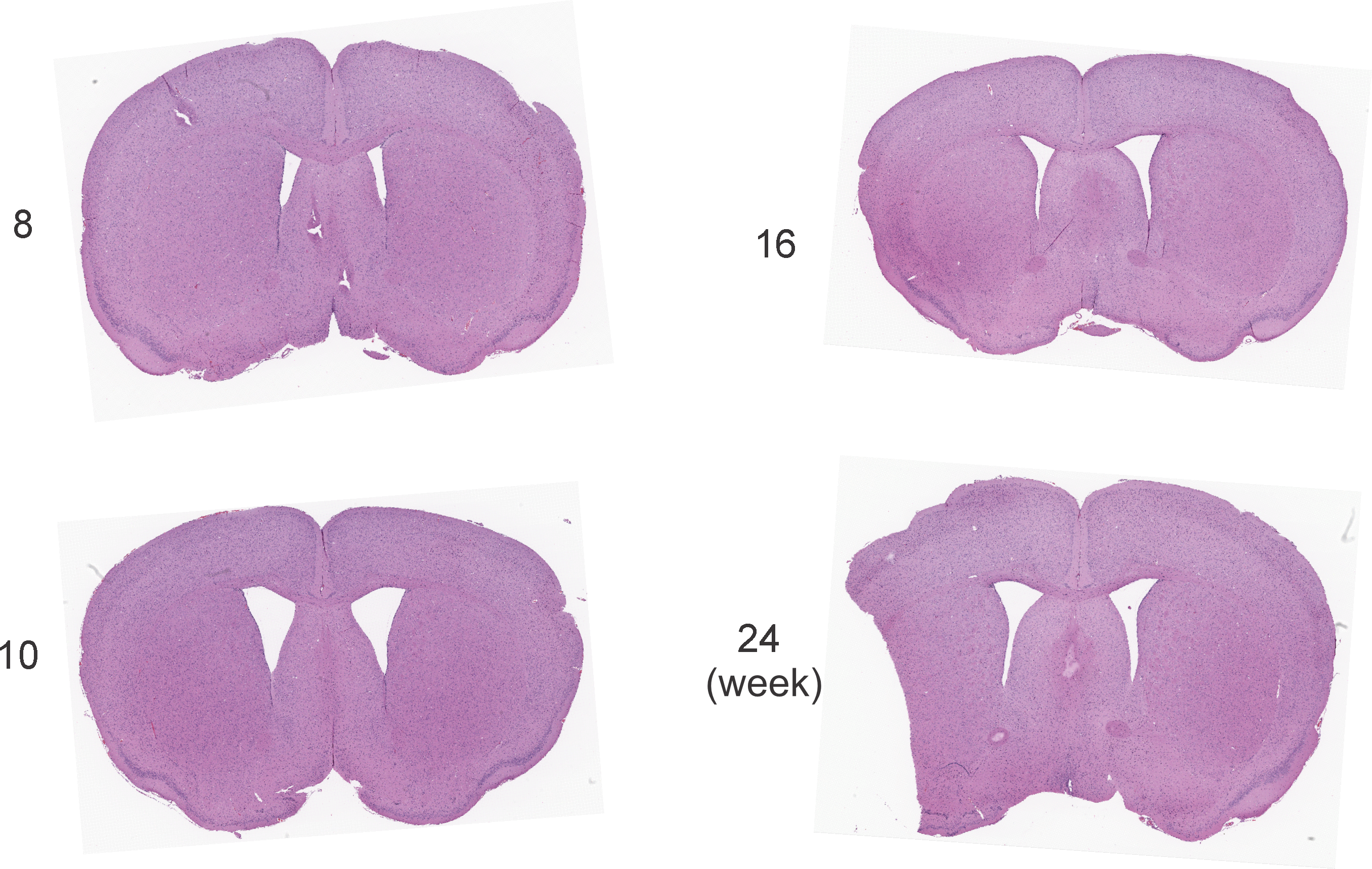

Supplement: Supplementary file 1 — Additional file 1: Figure S1. Brain sections at the level of the striatum, corpus callosum and motor cortex stained with H&E from animals in various ages as shown on the left of the images. There were no findings from any regions shown. [file 42826_2021_100_MOESM1_ESM.tif]

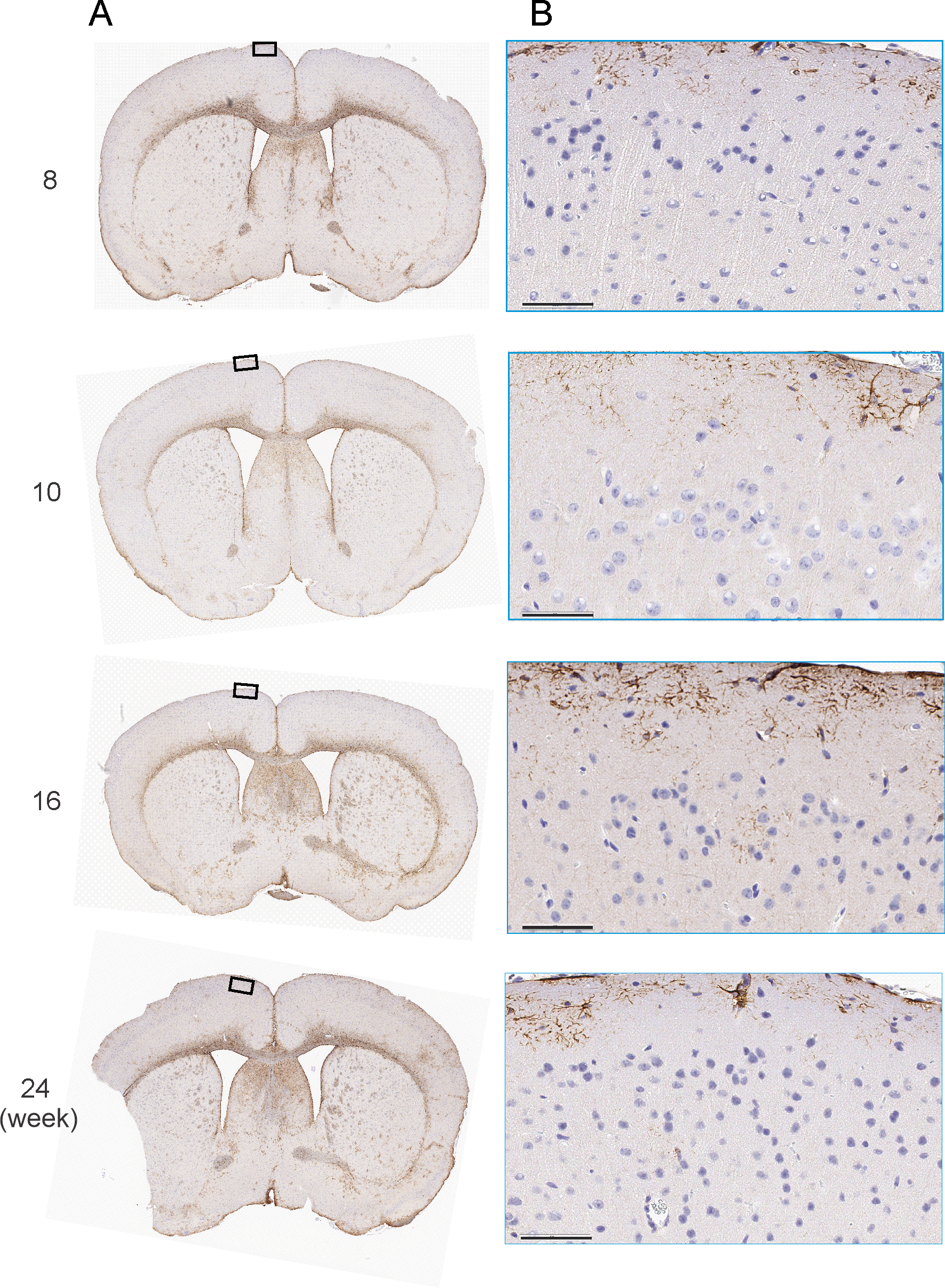

Supplement: Supplementary file 2 — Additional file 2: Figure S2. Brain sections at the level of the striatum, corpus callosum and motor cortex. (A) representative IHC images of GFAP immunoreactivity in adult male CBA/J mouse forebrain. No increased GFAP reactivity was observed in any of the areas mentioned above from mice of any age. (B) Magnified IHC images of the corresponding boxed areas in the cortex (A). Unlike the GFAP expression in the cerebellum, no increased GFAP expression was observed in the superficial layers of the cortex at age of week 24. Scale bar = 60 µm. [file 42826_2021_100_MOESM2_ESM.tif]
